# Tailoring care, advancing justice: predictors of forensic and legal engagement in survivors of sexual violence

**DOI:** 10.1186/s13584-025-00697-1

**Published:** 2025-06-23

**Authors:** Shani Yaakobi-Zelnik, Maya Peled Raz, Ateret Gewirtz-Meydan

**Affiliations:** 1 Bnai Zion Medical Center, Haifa, Israel; 2 Hebrew University of Jerusalem, School of Social Work and Social Welfare, Jerusalem, Israel; 3 University of Haifa, Haifa, Israel

**Keywords:** Sexual assault, Forensic examination, Police complaint, Sexual assault referral centers (SARC), Forensic examination, Trauma-informed care, Interdisciplinary care, Retrospective cohort study

## Abstract

**Background:**

One of the primary challenges faced by survivors of sexual abuse and assault is the fragmented nature of care. This begins immediately after the assault and continues throughout their recovery journey. To address this, specialized sexual assault referral centers (SARC) were established in Israel, providing comprehensive medical, legal, and emotional support. This study examines the association between these centers and survivors' consent to forensic examination and filing of police complaints.

**Methods:**

This retrospective cohort study analyzed data from 616 cases of sexual abuse and assault reported at the SARC at Bnai Zion Medical Center between January 2016 and May 2022. Data included demographic information, assault characteristics, treatment details, and survivors' consent to forensic examination and police complaint filing.

**Results:**

Four distinct profiles of sexual assault survivors were identified. The first profile included survivors who consented to both forensic examination and police complaints. This group was characterized by shorter intervals between the assault and arrival at the center, being accompanied by friends or family, more often agreeing to receive medication and experiencing more incidents involving more than one attacker. The second profile included survivors who consented to forensic examination only, and it consisted mainly of young women who arrived unaccompanied, later after the assault, expressed more willingness to receive medications and often involved single perpetrator assaults with alcohol use. The third profile involved survivors who filed only a police complaint and were characterized by lower rates of alcohol use during the assault and less frequent receipt of medication. The fourth profile comprised survivors who refused both forensic examination and police complaint, primarily young females who arrived unaccompanied and often reported experiencing forced vaginal or anal penetration. Key predictors of group membership included time since the assault, biological sex, assault type, alcohol use, medication, presence of an informal escort, and multiple perpetrators.

**Conclusions:**

The study underscores the importance of a victim-centered, trauma-informed approach to care, highlighting the need for tailored interventions to address the specific needs and barriers of each survivor profile. The findings suggest that timely access to medical care and supportive services is significantly associated with survivors' decisions to consent to forensic examinations and file police complaints.

**Supplementary Information:**

The online version contains supplementary material available at 10.1186/s13584-025-00697-1.

## Introduction

One of the main challenges faced by survivors of sexual abuse and assault is the fragmented nature of care they experience immediately after the assault and throughout their recovery journey. Research underscores the profound trauma experienced by survivors in the aftermath of sexual assault, emphasizing the critical impact of immediate post-incident therapy processes centralized in one place [4].

To provide survivors with holistic care that includes medical, legal, and emotional support without external referrals for initial assistance, specialized sexual assault referral centers (SARC) were established in Israel, integrating the response immediately after the assault. Currently, there are eleven SARCs in Israel, located in different public hospitals that provide an immediate response to sexual abuse and assault [12]. These centers embody a groundbreaking multidisciplinary integrated model that seamlessly combines medical attention, psychological support, and forensic examination, addressing the diverse needs of sexual abuse and assault survivors promptly and compassionately. Professionals from various disciplines, including physicians, forensic medicine, social work, and nursing collaborate to provide holistic care, acknowledging the multifaceted consequences of sexual assault [13, 24, 25]. This model is representative of a global trend towards immediate response and cohesive care for sexual assault victims, a practice backed by considerable literature showcasing its efficacy in both improving patient outcomes and gathering prosecutable evidence [4, 18].

Medically, the centers administer medications to prevent infections, sexually transmitted diseases, and unintended pregnancies and provide treatment for injuries related to the assault. Emotionally, they provide crisis intervention as well as construct post-release treatment and follow-up plans, including referrals for additional community-based treatment [13]. In the legal realm, the SARC conducts forensic evidence collection, involving DNA samples, semen, or skin cells, utilizing specialized equipment.

The SARC's streamlined centralized care approach not only potentially increases victims' likelihood of consenting to a forensic examination but also provides an immediate, supportive environment to address their pressing needs [13] The significance of a compassionate initial response is highlighted, positively influencing victims' engagement with both legal and healthcare services.

The timing of treatment is pivotal, with evidence suggesting that a prompt forensic examination is most effective [25]. Centralized care at SARCs ensures timely access, encouraging victims to undergo examinations promptly. This not only enhances the quality of evidence collected but also exerts a positive influence on legal outcomes. In addition, effective communication between healthcare providers and law enforcement emerges as another critical factor in fostering victim cooperation [24]. Centralized care centers, through their proximity and specialists' collaborative approach, foster a positive environment that reassures victims and cultivates trust in the entire process. Lastly, immediate psychological support during the initial treatment phase addresses emotional barriers to consent. Victims grappling with feelings of shame, guilt, or fear of disbelief find solace in the supportive and non-judgmental environment provided in the SARC.

Another gap in the literature relates to how reporting to the police is related to recovery processes after sexual assault. Research underscores both potential benefits and significant risks, with psychological outcomes depending heavily on the quality of interactions and systemic practices. Positive experiences, such as supportive and respectful interactions with law enforcement, have been linked to improved mental health outcomes and greater satisfaction with the legal process. Conversely, negative experiences with the legal system, including secondary victimization—a phenomenon where survivors feel blamed, dismissed, or retraumatized during interactions with law enforcement—are frequently reported [17, 20]. SARCs, through their multidisciplinary approach, may help mitigate such risks by providing a trauma-informed and survivor-centered framework that minimizes the likelihood of secondary victimization.

The incorporation of medical, psychological, and legal services within this model creates an environment where survivors may feel more informed, supported, and empowered to engage in the forensic examination process. The convergence of medical treatment, psychological assistance, and legal support within a singular setting not only reflects internationally recommended best practices recommended in international literature but is also an embodiment of victim-centered care, a concept that prioritizes the dignity, autonomy, and holistic well-being of survivors [24].

## The current study

Informed by global and Israeli literature, the study aims to provide a comprehensive understanding of the demographic, clinical, and situational factors of sexual assault survivors treated at a SARC. Additionally, it seeks to explore patterns of consent to forensic examination and filing police complaints among the survivors. This research holds significant clinical implications, offering insights into the SARC population and the motivations that influence survivors' willingness to undergo forensic examination and potentially engage with law enforcement. Addressing this literature gap promises to contribute valuable knowledge to the field. The study incorporates a retrospective cohort investigation, analyzing data from the records of all patients treated at the SARC at Bnai Zion Medical Center between January 2016 and May 2022. Commencing with the date of initiation of systematic data collection at the center, the study concludes with the implementation of Amendment 15 to the Crime Victims’ Rights Law, which hindered the ability to track whether survivors filed a police complaint. Enacted in May 2022, the amendment mandated that all forensic kits be transferred directly to the police's national logistics center. Previously, SARCs stored the kits and were notified upon police collection, enabling tracking. Under the new system, SARCs no longer receive such notifications, preventing further data collection. Demographic information, assault characteristics, provided treatment, patient consent or refusal for forensic evidence collection, and whether a police complaint was issued by the victim were all coded without patient identification, following approval from the Bnai Zion Medical Center Research Ethics Committee [BNZ-0059–23] and the University of Haifa research ethics committee [244/23].

## Methods

### Participants and procedure

The study analyzed all cases of sexual abuse and assault reported at the SARC at Bnai Zion Medical Center in Israel between January 2016–May 2022. The sample included all patients treated at the center during this period. This specific date range was chosen because January 2016 marks the beginning of systematic computerized documentation of patients, while May 2022 corresponds to the enactment of Amendment 15 to the Crime Victims’ Rights Law, as explained above.

Each case was analyzed for demographic details, such as age, sex, marital status, and ethnicity. Timing and circumstances of the assault were also considered, including the arrival time at the center and the time elapsed since the assault. Psychological and medical history included previous assaults, prior emotional therapy, and any psychiatric background. Living arrangements, such as whether the individual resided in an institutional setting, were noted. Details of the assault were also examined, including the relationship with the perpetrator(s), type of assault, and involvement of multiple perpetrators. Substance use information included drug and alcohol use, as well as any suspicion of GHB (Gamma-hydroxybutyrate, commonly known as a “date rape drug”) exposure. Additionally, the presence of support, such as whether the individual was escorted by someone and the referral source, was recorded. Treatment information documented the gender of the attending physician, medications administered, and any referrals for emotional therapy.

### Data analysis

The sample suffers from 4.49% of data missing with 53 distinct patterns of absence. Jamshidian and Jalal’s non-parametric Missing Completely At Random (MCAR) test indicated the data were missing at random (MAR): i.e., Hawkins’ test [*χ*^2^_(10)_ = 390.74, *p* = 8.79^–78^] and Anderson–Darling rank test [*T*_median_ = 10.00, *p* = 0.003] were significant. We used the Multiple Imputation procedure [21] with 50 complete datasets to handle the missing data using the *mice* R package. Analyses were then conducted on these datasets, and the reported results are the pooled outcomes of these analyses.

Next, we classified the cases based on two dichotomous variables: consent to forensic examination (yes, no) and filing a police complaint (yes, no). Overall, 334 consented to forensic examination and filed a police complaint, 147 only consented to forensic examination, 69 only filed a police complaint, and 66 did not consent to an examination nor filed a police complaint. The classification of cases into these four groups (+ + ,- + , + -,–) was guided by the primary research focus on the relationship between consent to forensic examination and the decision to file a police complaint. These groups were pre-determined as they represent the possible combinations of these two key decisions, enabling an exploration of whether distinct profiles could be identified. It was not assumed in advance that significant differences would necessarily emerge between the groups. To distinguish between these groups in a series of background measures, we followed these two steps: We began by examining the differences in each of the background measures by a series of Kruskal–Wallis tests for the ordinal measures (age group, arrival time at the center, and time since the assault), and chi-square for the independence of measures for the categorical measures (biological sex, marital status, children, previous assault, previous emotional therapy, psychiatric background, dwelling in an institution, ethnicity, relationship to perpetrators, assault type, multiple perpetrators, drug use, alcohol use, suspicion of GHB exposure, escorted by, referred by, attending physician gender, medication, and referral for emotional therapy); Posthoc-analyses were Dunn’s tests with holm adjustment, and residuals of chi-squared tests with a 5% False Discovery Rate adjustment for the Kruskal–Wallis and chi-square tests respectively. In the second step, we selected all significant variables from the first step and inserted them as predictors in a multinomial ordinal regression model in which the outcome measure was the study group. This analysis enabled us to assess each predictor’s relative and unique contribution in determining the likelihood of survivors being members of a specific study group. The analysis was performed using the *nnet* R package and the *multinom* function, and the *effects* R package for visualization.

## Results

The sample comprises 616 cases, with primarily female survivors (90%), with males making up 9.5% and a minority identifying as other genders (0.6%). Of the survivors 82% were single and just a minority were married (4.5%). The majority of survivors were aged 18–29 (54%), followed by those over 30 (23%) and those aged 14–17 (16%). Most survivors identified as Jewish (78%), with 19% identifying as Arab. A significant minority (20%) of the sample resided in an institution. Regarding the nature of the assaults, half of the recorded assaults (56%) involved a perpetrator who was known to the survivor before. In 14% of cases, survivors reported multiple perpetrators. Alcohol was involved in 26% of assaults.

Nearly two-thirds (64%) of survivors reached the center within the initial 24-h period after the assault. Informal escort—of family members or friends, was observed in 51.5% of the cases, while 34% had formal escorts – referring to professionals such as social workers, police officers, or institutional staff. Medication was administered to 60% of survivors. Most of the men treated in the SARC, and thus included in the sample, were under guardianship and reside in institutional dwellings, for whom decisions were made by their attendants or legal guardians.

The study identified four distinct profiles of sexual assault survivors: More than half of the survivors (54%) consented to a forensic examination and filed a police complaint; 24% consented to forensic examination only; 11% filed only a police complaint, and 11% did not consent to forensic examination or police complaint.

Full descriptive statistics are presented in Supplementary File 1.

### Step 1: differences in study measures according to behavioral groups

When looking at the four distinctive identified groups, differences were found in the time since the assault, biological sex, ethnicity (Jewish, Arabic), assault type, multiple perpetrators, drug use, alcohol use, escorted by, referred by, and medication. Significant results were plotted and presented in Supplementary File 2 alongside the tests’ statistics.

We found that the group who consented to forensic examination and filed a police complaint experienced significantly shorter intervals between the assault and arrival at the center (all *p* < 0.05), the lowest frequency of arriving without an escort (all *p* < 0.05) and the highest frequency of Arab ethnicity (all *p* < 0.05). In addition, all who consented to forensic examination (i.e., both those who consented to examination and filed a complaint and those who only agreed to the examination) had significantly higher prescription of medications within the SARC treatment than those who did not consent to the examination (which is sync with the consent to the whole medical procedure (all *p* < 0.05). Conversely, all who filed a police complaint had a higher frequency of being male (all *p* < 0.05), lower frequency of vaginal penetration (which is in sync with the higher frequency of men; all *p* < 0.05), and lower frequency of arriving without an escort (all *p* < 0.05). Finally, the group that tended not to consent to a forensic examination nor to filing a police complaint had the highest frequency of multiple perpetrators (all *p* < 0.05), and drug and alcohol use (all *p* < 0.05).

### Step 2: multinomial ordinal regression model

Results are presented in Supplementary File 3. We found eight unique and significant predictors of group membership. Time since the assault: The model indicated that the longer the time interval between the assault and the arrival at the center, the lower the likelihood of consenting to a forensic examination and filing a police complaint and the higher the likelihood of only consenting to a forensic exam without filing a complaint (see Fig. 1 in Supplementary File 3). Survivors’ biological sex: Being male correlates with increased likelihood of consenting to both a forensic examination and filing a police complaint. Female survivors were more likely to consent only to a forensic exam or to decline both the examination and the filing of a complaint. Male and female survivors were equally likely only to file a police complaint (see Fig. 2 in Supplementary File 3). Biological sex itself may not be the primary predictor of these behaviors. Rather, the increased likelihood of filing a complaint is more strongly associated with individuals living in institutional settings who are brought for examination by institution staff. In our center, this demographic largely consists of male survivors. This institutional factor, rather than biological sex alone, appears to be the more significant predictor of complaint filing and forensic examination consent. Multiple perpetrators: Multiple perpetrators significantly increased the possibility of consenting to a forensic exam and filing a police complaint while decreasing the probability of consenting only to a forensic exam (see Fig. 2 in Supplementary File 3).

Assault type: Forced vaginal and/or anal penetration significantly decreased the likelihood of consenting to a forensic examination and filing a police complaint, while increased the possibility of avoiding both (see Fig. 3 in Supplementary File 3). Alcohol use and medications: Alcohol use significantly decreased the likelihood of filing a police complaint while increasing the probability of consenting to a forensic examination; use of medications significantly reduced the possibility of only filing a police complaint while increasing the likelihood of consenting to a forensic exam or to both (i.e., an examination and filing a complaint; see Fig. 4 in Supplementary File 3). Escort: Informal escort significantly increased the likelihood of consenting to a forensic exam and filing a police complaint while decreasing the probability of consenting only to a forensic exam or avoiding both. Informal escort did not change the likelihood of only filing a police complaint (see Fig. 5 in Supplementary File 3). In summary of the Multinomial Ordinal Regression Model (MORM), the present study reveals four distinct profiles of sexual assault survivors based on their decisions regarding forensic examination and police reporting,

## Discussion

The current study used a sample of 616 cases of sexual assault survivors who attended SARCs in Israel, to examine the predictors of forensic examination consent and police complaint filing. We divided them into four profiles that offer valuable insights into the factors that might be influencing survivors' choices in the aftermath of assault. By understanding the unique characteristics and needs of each group, service providers and policymakers can develop tailored interventions to better support survivors and improve outcomes across the continuum of care illustrated in Table 1.

**Table 1:**
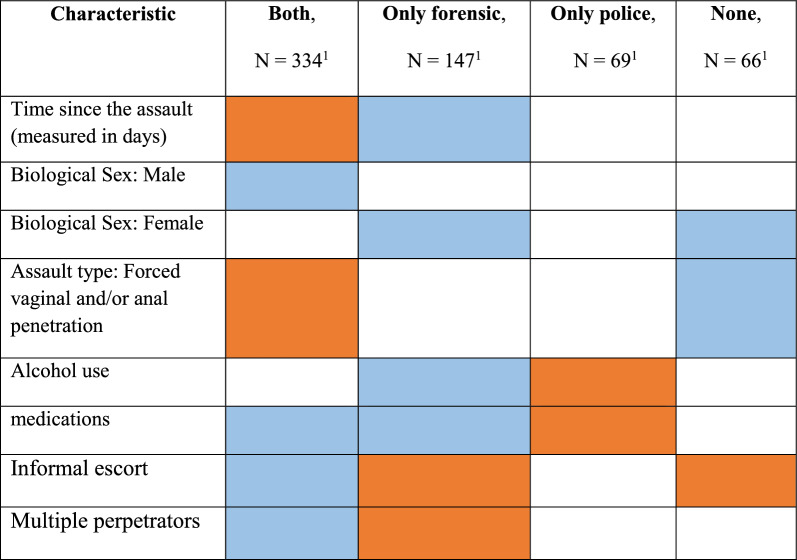
Likelihood of being a member of each study group

Blue- Higher likelihood of being a member of study group.

Orange- Lower likelihood of being a member of study group.

### Profile 1: consent to forensic examination and filing a police complaint

This group of survivors presents cases that align with what is often a more widely recognized pattern of sexual assault (rape), which is often associated with a higher tendency to report and seek help [6]. These survivors span a diverse age range and are predominantly female, who arrived at the SARC shortly after the attack, often accompanied by friends or family members. It can be assumed that these are survivors who disclosed the assault at an early stage and received support from family and friends, which in turn suggests a strong support system and the ability to take proactive action to consent to the medical treatment, forensic examination and file a complaint with the police [2, 8, 15, 22, 23]. These distinctions highlight the importance of considering contextual factors, such as living arrangements and support systems when analyzing patterns in reporting and consent for forensic examinations among sexual assault survivors. It underscores the complex interplay of various factors influencing survivors' decisions and experiences in the aftermath of assault**.**

Men were significantly more prevalent in this group. This is not surprising, as most of the men treated in the SARC, and thus included in the sample, were under guardianship and reside in institutional dwellings, In such cases, the pathway to receiving care is often mediated by a guardian or caregiver, who not only facilitates access to the SARC but also initiates the formal reporting process. This context may partially explain the overrepresentation of men under guardianship in the data, and specifically in this group. However, this pattern raises critical concerns about the invisibility of other groups of men who may be equally, if not more, vulnerable to sexual victimization but lack a comparable support system to navigate access to care. Specifically, men who are not institutionalized, such as those in the LGBTQ + community, migrants, or unhoused individuals that may face multiple barriers to accessing SARCs.

It should be noted that our findings align with data from the Association of Rape Crisis Centers in Israel, which indicate that only 13% of those who contacted the centers in 2025 were male individuals—adults, adolescents and children. According to the Association, this figure does not necessarily reflect the actual prevalence of sexual assault among males but rather points to significant underreporting. This underreporting is attributed, among other factors, to social stigma and gendered expectations that hinder males from recognizing themselves as victims and from seeking help [1].

### Profile 2: consent to forensic examination only

This group of survivors, who chose to consent solely to a forensic examination, illustrates a complex and nuanced response following an assault, characterized by a sense of ambivalence. As mentioned, these survivors typically had a longer time interval between the assault and SARC arrival compared to the other groups, they tended to expressed more willingness to receive medications which may indicate that the main motivation of this group of survivors was to receive medical treatment. The assaults in this group typically involved a single perpetrator and the use of alcohol—variables that, as observed in previous studies, are linked to a sense of shame and guilt and contribute to a reduced likelihood of engaging with the police [10, 15]. Combining the finding that these survivors were mostly young women who arrived unaccompanied, it highlights the increased difficulty they face, as the experience of loneliness and shame often accompanying sexual assault survivors may further act as a barrier to filing a police report [5, 9].

As mentioned above, the characteristics of the current profile may indicate an ambivalent position about receiving formal help. On the one hand, late arrival could be due to fear of negative reactions from the service providers [26]. On the other hand, consent to forensic examination might have been influenced by the supportive and compassionate care they received from the staff during their visit to the SARC, potentially leaving the door open for future criminal proceedings. Another source of ambivalence in the current group may be the experience of trust. Trust in the health care system leads them to use formal support [14] such as SARC and agree to a forensic evaluation, in the face of lack of trust in the police, that at this stage leads them to avoid filing a complaint [11].

### Profile 3: file police complaint only

The group that only filed a police complaint presents unique characteristics; male and female survivors were equally likely to fall into this category. Their assaults typically involved lower alcohol use and they were less likely to agree to medication. The equal representation of male and female survivors in this group may be explained by distinct factors. For male survivors, particularly those declared incompetent, which are most of the men in this sample, their inclusion in this group may reflect the inherent challenges associated with conducting physical examinations on individuals with hindered understanding and lack of ability to consent to the procedure. These difficulties could lead to guardian's preference for filing a police complaint only.

For female survivors, the decision to file a complaint without consenting to forensic examination may stem from various considerations. One possibility is the belief that gathering forensic evidence may be impossible, leading them to focus solely on the legal process. This decision might be influenced by the timing of the report or the circumstances surrounding the assault, which may have diminished the perceived likelihood of finding conclusive forensic evidence. Another plausible explanation relates to the existing knowledge in the literature about the experiences of forensic evidence collection, which is often described as intrusive, humiliating, and re-traumatizing [16]. The decision of this group to avoid such an examination may reflect a desire to protect themselves from further emotional or physical distress. However, this choice does not necessarily indicate a lack of interest in seeking justice,rather, it may represent a calculated decision to achieve justice through means that feel less personally invasive.

### Profile 4: no forensic examination or police complaint

The group that did not undergo forensic examination or file a police complaint, faced particular challenges. The demographic of young females, who arrived unaccompanied after facing sexual assaults involving forced penetration underscored potential barriers to seeking legal and medical help. The decision to forgo examination and formal complaint may have stemmed from fear, concerns about privacy, or distrust of legal processes, aligned with prior findings [3, 10, 15]. Survivors may also have experienced feelings of guilt and fear of judgment, influenced by negative past interactions with law enforcement [2, 3, 7, 11]. Additionally, the lack of a support system, formal or informal, further isolated the survivors from seeking legal assistance. It appeared that their arrival at the SARC without utilizing legal or medical avenues may signify their need for emotional support and validation from professionals who understood the complexities of sexual assault, highlighting a need to process these emotional experiences.

### Clinical and policy implications

The findings of this study underscore the importance of a victim-centered, trauma-informed approach that acknowledges the diverse and complex realities of sexual assault survivors. The identification of distinct profiles is not intended to establish a rigid typology or to assign survivors to predetermined treatment tracks. Rather, it serves to highlight the heterogeneity among survivors and to emphasize that individuals respond differently to trauma and to the services available to them. These profiles reveal patterns that can help practitioners better understand the varied needs, barriers, and motivations of survivors—without reducing them to fixed categories.

For example, since the majority of the sample consisted of young, single, Jewish women who utilized SARC services, tailoring care to this profile might include culturally sensitive communication strategies, gender-specific training for healthcare providers, and specialized support services that address the experiences and expectations of this group. The analysis also found that survivors who arrived alone and reported alcohol use during the assault were less likely to file a police complaint, suggesting that shame or fear of blame may influence their help-seeking behaviors. In such cases, trauma-oriented medical care that actively avoids victim-blaming and addresses stigma related to substance use can help validate survivors’ experiences and restore their sense of control.

Tailoring interventions in this way can enhance both survivors’ trust in services and their willingness to engage with medical and legal processes. The goal is not to “fit” each survivor into a category, but rather to equip care teams with a broader understanding of survivor variability—thereby promoting more responsive, respectful, and effective care.

Second, the study highlights the importance of timely access to medical care for survivors seeking assistance after an assault. As indicated by the findings, survivors tend to seek medical care more than filing a police complaint as time passes after the assault. Given that SARCs typically provide care within the first week post-assault, survivors seeking assistance beyond this timeframe may face challenges in accessing appropriate support services. Therefore, policymakers should consider expanding training initiatives to include gynecologists and family physicians in the community, as they are often the first point of contact for survivors seeking medical assistance after the initial week. These healthcare professionals should be trained to recognize signs of trauma, provide supportive care, and make appropriate referrals, so the healthcare system can better meet the needs of survivors throughout their recovery journey.

Third, the segmentation of the patient population at SARCs, as identified in the current analysis as well as in Mizrachi et al.[19], shows that young, single, Jewish women are the primary users. This raises important questions regarding public awareness of SARCs, particularly among underserved groups. To address this, targeted awareness campaigns and a national media strategy should be implemented, utilizing traditional and digital platforms to promote SARC services. Messaging should be culturally sensitive, tailored to diverse groups including minorities, men, and individuals of various marital statuses, and delivered through social media, community centers, and faith-based organizations. Additionally, as only 10% of those treated in the SARC identified as male or other, and most male survivors in the sample were institutionalized and under guardianship, efforts are needed to improve outreach to other at-risk male populations, such as LGBTQ + men, who may be less likely to access SARC services. Outreach in educational institutions is also crucial, with school and university programs designed to inform young adults about SARCs and their inclusive support. Community engagement should involve collaboration with leaders, NGOs, and advocacy groups to build trust and awareness among underrepresented populations. Finally, continuous data collection and analysis are essential to monitor the effectiveness of outreach efforts and identify service gaps.

## Limitations and future research directions

The present study was conducted on data from one SARC in Israel, therefore, the sample's geographic and cultural specificity may limit the generalizability of the findings. The geographic and cultural specificity limits applicability to other regions and cultures. The low representation of male survivors and certain ethnic groups may skew results, and reliance on self-reported data introduces potential biases. The lack of longitudinal data restricts insights into long-term outcomes, and the scope of variables is limited, omitting factors like socio-economic status and sexual orientation. As part of this, at this stage, it is not possible to assess the relationship between the choice to file a police complaint and consent to forensic examination, and the long-term recovery processes after sexual assault. Additionally, the study did not account for potential confounding variables such as levels of PTSD and dissociation, or variables related to satisfaction with the treatment and the caregivers, which are important factors in understanding survivors' experiences.

Future research should encompass diverse regions, incorporate a wider array of variables, and employ longitudinal methodologies to offer a more comprehensive understanding of survivors' help-seeking behaviors. Additionally, future research should continue to explore these profiles in greater depth, potentially identifying additional sub-groups or intersectional factors that influence help-seeking behaviors and recovery processes. The association between childhood sexual trauma and these profiles should also be explored, as this connection may provide valuable insights into the dynamics influencing survivors' behaviors and recovery processes. In addition, longitudinal studies examining the long-term impact of tailored interventions on victims' well-being, engagement with the criminal justice system, and overall recovery would further inform evidence-based practices in this area. Finally, comparative studies across diverse cultural and socioeconomic contexts could shed light on the role of systemic factors in shaping victim experiences and help-seeking patterns.

## Conclusions

This study provides insights into the diverse profiles and decision-making processes of sexual assault victims regarding forensic exams and police reporting. By identifying four distinct profiles, based on victims' consent and reporting choices, the findings reveal the heterogeneity of this population and the varied factors influencing their help-seeking behaviors. Key factors such as time elapsed since the assault, presence of escorts, and substance use emerged as important influences across profiles. The study underscores that tailored, victim-centered interventions accounting for each profile's unique needs and barriers are crucial for improving outcomes. These conclusions offer actionable recommendations for policymakers to improve SARC services in Israel by addressing the specific needs of diverse survivor groups. Future research should explore additional sub-groups, assess the longitudinal impacts of tailored interventions, and conduct cross-cultural comparisons. A coordinated, multidisciplinary approach involving various stakeholders is essential to building a comprehensive continuum of care that empowers survivors, supports healing, and improves access to justice.

## Supplementary Information

Additional file 1.

Additional file 2.

Additional file 3.

## Data Availability

The datasets generated during the current study are available from the corresponding author on reasonable request.

## Abbreviations

SARC: Sexual assault referral centers
