# Supplementary material for: Tailoring care, advancing justice: predictors of forensic and legal engagement in survivors of sexual violence
Source: Isr J Health Policy Res. 2025 Jun 23;14:38. doi: 10.1186/s13584-025-00697-1 (PMC12183890; doi:10.1186/s13584-025-00697-1)
Supplement: Supplementary file 1 — Additional file 1. [file 13584_2025_697_MOESM1_ESM.docx]

| **Characteristic** | **Only forensic**, N = 147^1^ | **Both**, N = 334^1^ | **None**, N = 66^1^ | **Only police**, N = 69^1^ |
| --- | --- | --- | --- | --- |
| Biological Sex |  |  |  |  |
| Men | 8 (5.4%) | 41 (12%) | 1 (1.5%) | 9 (13%) |
| Women | 139 (95%) | 293 (88%) | 65 (98%) | 60 (87%) |
| Age Group |  |  |  |  |
| < 10 | 2 (1.4%) | 19 (5.7%) | 1 (1.5%) | 3 (4.3%) |
| 10-13 | 2 (1.4%) | 12 (3.6%) | 0 (0%) | 3 (4.3%) |
| 14-17 | 19 (13%) | 59 (18%) | 8 (12%) | 11 (16%) |
| 18-29 | 100 (68%) | 162 (49%) | 41 (62%) | 29 (42%) |
| 30 + | 24 (16%) | 82 (25%) | 16 (24%) | 23 (33%) |
| Marital Status |  |  |  |  |
| Not Married | 143 (97%) | 318 (95%) | 62 (94%) | 65 (94%) |
| Married | 4 (2.7%) | 16 (4.8%) | 4 (6.1%) | 4 (5.8%) |
| Children |  |  |  |  |
| No | 129 (88%) | 269 (81%) | 54 (82%) | 58 (84%) |
| Yes | 18 (12%) | 65 (19%) | 12 (18%) | 11 (16%) |
| Previous Assault |  |  |  |  |
| No | 90 (61%) | 220 (66%) | 39 (59%) | 45 (65%) |
| Yes | 57 (39%) | 114 (34%) | 27 (41%) | 24 (35%) |
| Previous Emotional Therapy |  |  |  |  |
| No | 77 (52%) | 156 (47%) | 26 (39%) | 27 (39%) |
| Yes | 70 (48%) | 178 (53%) | 40 (61%) | 42 (61%) |
| Psychiatric Background |  |  |  |  |
| No | 105 (71%) | 217 (65%) | 42 (64%) | 42 (61%) |
| Yes | 42 (29%) | 117 (35%) | 24 (36%) | 27 (39%) |
| Dwelling in Institution |  |  |  |  |
| No | 123 (84%) | 269 (81%) | 52 (79%) | 50 (72%) |
| Yes | 24 (16%) | 65 (19%) | 14 (21%) | 19 (28%) |
| Ethnicity |  |  |  |  |
| Jewish | 128 (87%) | 255 (76%) | 54 (82%) | 58 (84%) |
| Arabic | 19 (13%) | 79 (24%) | 12 (18%) | 11 (16%) |
| Relationship to Perpetrator |  |  |  |  |
| Acquaintance | 53 (36%) | 114 (34%) | 17 (26%) | 19 (28%) |
| Family Member | 3 (2.0%) | 12 (3.6%) | 3 (4.5%) | 3 (4.3%) |
| Partner | 9 (6.1%) | 27 (8.1%) | 4 (6.1%) | 4 (5.8%) |
| Stranger | 82 (56%) | 181 (54%) | 42 (64%) | 43 (62%) |
| Assault Type |  |  |  |  |
| Forced Anal Penetration | 21 (14%) | 63 (19%) | 6 (9.1%) | 14 (20%) |
| Forced Vaginal Penetration | 122 (83%) | 246 (74%) | 55 (83%) | 48 (70%) |
| Unwanted Sexual Contact | 4 (2.7%) | 25 (7.5%) | 5 (7.6%) | 7 (10%) |
| Multiple Perpetrators |  |  |  |  |
| No | 136 (93%) | 281 (84%) | 52 (79%) | 62 (90%) |
| Yes | 11 (7.5%) | 53 (16%) | 14 (21%) | 7 (10%) |
| Drug Use |  |  |  |  |
| No | 141 (96%) | 289 (87%) | 55 (83%) | 63 (91%) |
| Yes | 6 (4.1%) | 45 (13%) | 11 (17%) | 6 (8.7%) |
| Alcohol Use |  |  |  |  |
| No | 100 (68%) | 254 (76%) | 40 (61%) | 58 (84%) |
| Yes | 47 (32%) | 80 (24%) | 26 (39%) | 11 (16%) |
| GHB Use Suspicion |  |  |  |  |
| No | 124 (84%) | 294 (88%) | 54 (82%) | 63 (91%) |
| Yes | 23 (16%) | 40 (12%) | 12 (18%) | 6 (8.7%) |
| Escorted By |  |  |  |  |
| Formal Escort | 71 (48%) | 164 (49%) | 24 (36%) | 36 (52%) |
| Informal Escort | 28 (19%) | 104 (31%) | 7 (11%) | 14 (20%) |
| No Escort | 48 (33%) | 66 (20%) | 35 (53%) | 19 (28%) |
| Referred By |  |  |  |  |
| Self-Referral | 28 (19%) | 36 (11%) | 15 (23%) | 6 (8.7%) |
| Not Self-Referral | 119 (81%) | 298 (89%) | 51 (77%) | 63 (91%) |
| Arrival Time to the Center |  |  |  |  |
| 07:01-15:00 | 63 (43%) | 118 (35%) | 31 (47%) | 25 (36%) |
| 15:01-23:00 | 45 (31%) | 109 (33%) | 12 (18%) | 20 (29%) |
| 23:01-07:00 | 39 (27%) | 107 (32%) | 23 (35%) | 24 (35%) |
| Attending Physician Gender |  |  |  |  |
| Female Physician | 60 (41%) | 122 (37%) | 19 (29%) | 20 (29%) |
| Male Physician | 79 (54%) | 206 (62%) | 27 (41%) | 32 (46%) |
| No Attending Physician | 8 (5.4%) | 6 (1.8%) | 20 (30%) | 17 (25%) |
| Medication |  |  |  |  |
| No | 42 (29%) | 104 (31%) | 42 (64%) | 50 (72%) |
| Yes | 105 (71%) | 230 (69%) | 24 (36%) | 19 (28%) |
| Referral for Emotional Therapy |  |  |  |  |
| Already has therapy | 54 (37%) | 114 (34%) | 29 (44%) | 23 (33%) |
| Didn’t want a referral | 26 (18%) | 50 (15%) | 11 (17%) | 10 (14%) |
| Referred | 67 (46%) | 170 (51%) | 26 (39%) | 36 (52%) |
| Time Since the Assault | 3.07 (2.51) | 2.07 (1.59) | 3.06 (2.16) | 3.09 (3.04) |
| ^1^n (%); Mean (SD) | | | | |
