# Supplementary figures and images for: Tailoring care, advancing justice: predictors of forensic and legal engagement in survivors of sexual violence
Source: Isr J Health Policy Res. 2025 Jun 23;14:38. doi: 10.1186/s13584-025-00697-1 (PMC12183890; doi:10.1186/s13584-025-00697-1)

**Supplementary File 1 – Significant Differences Between Groups**


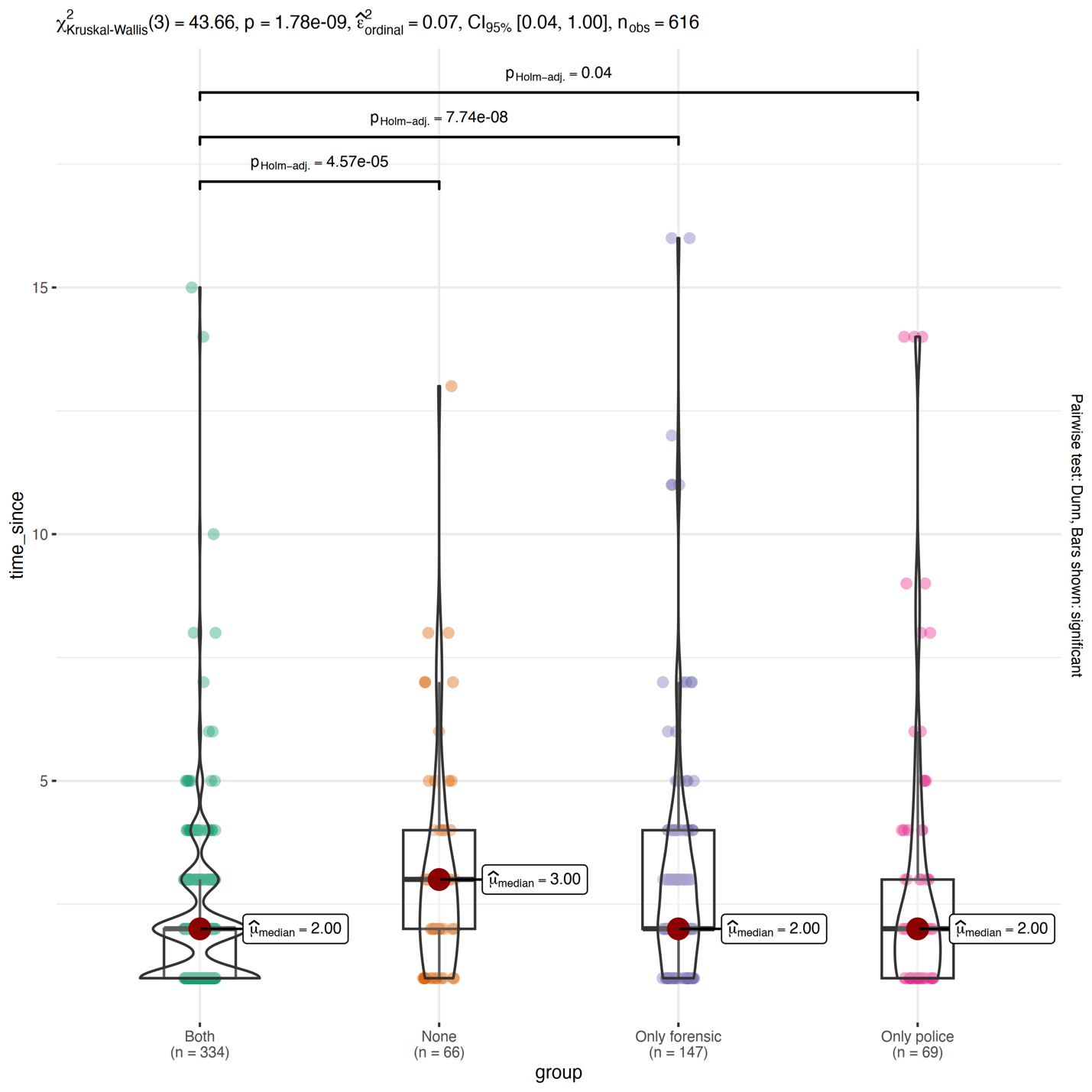


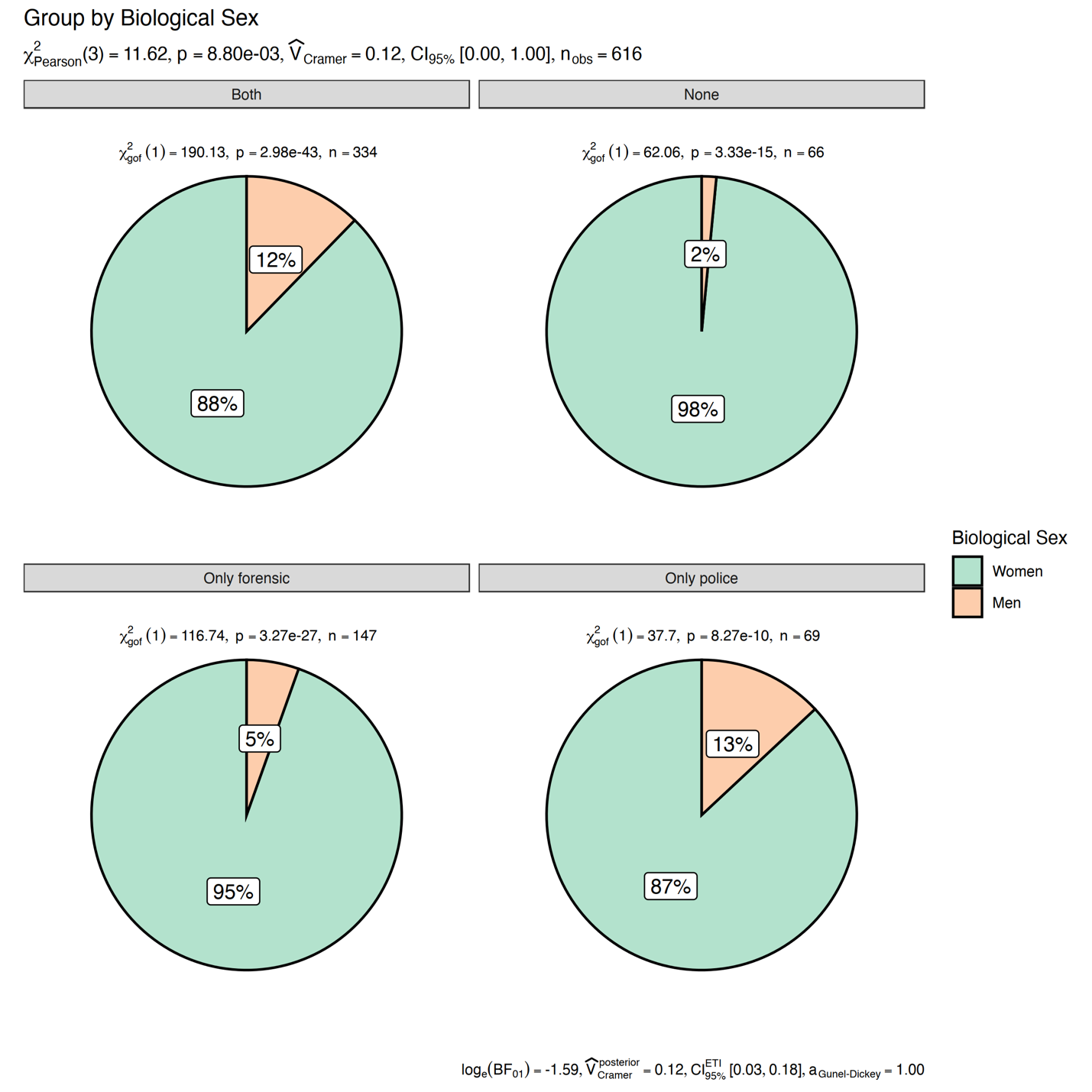


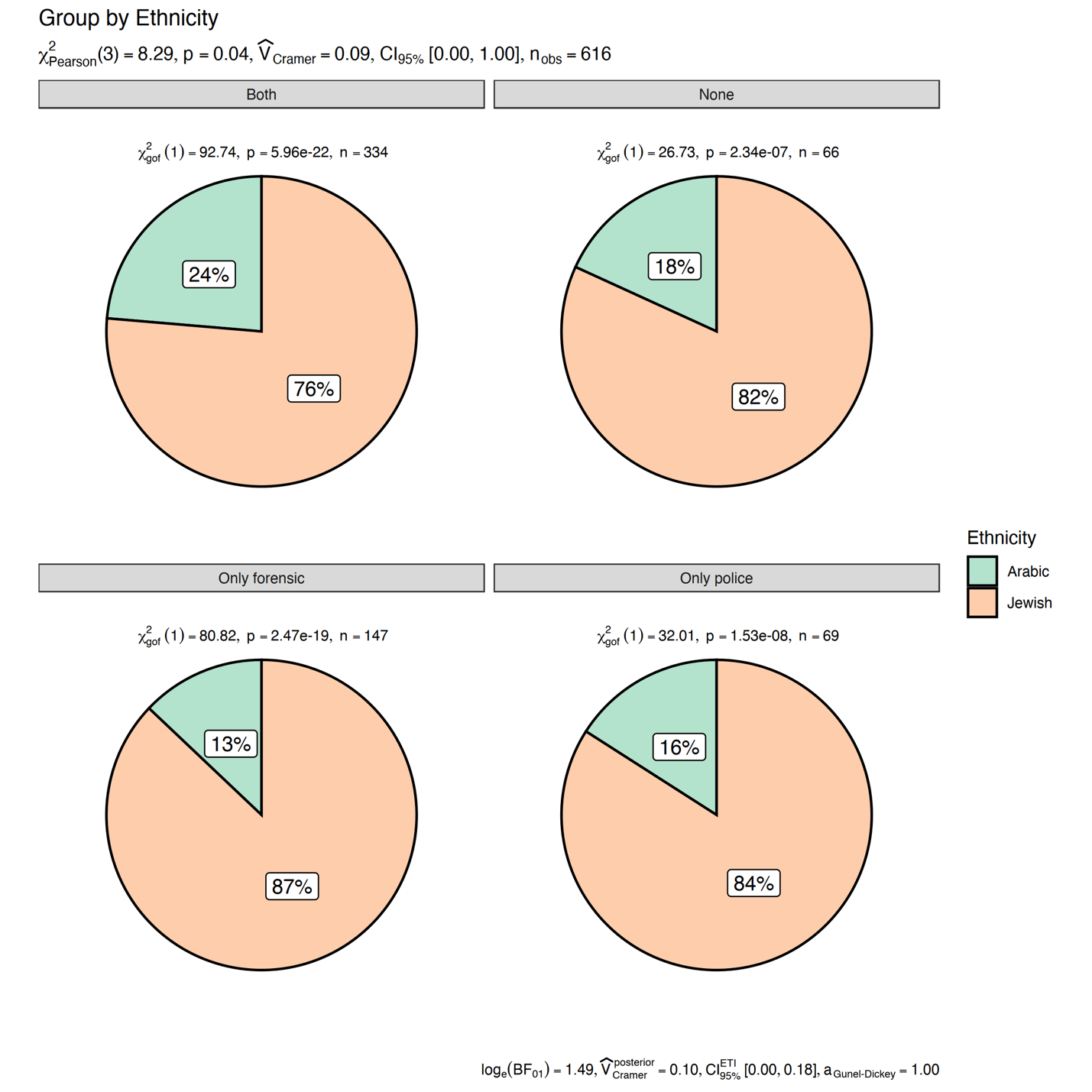


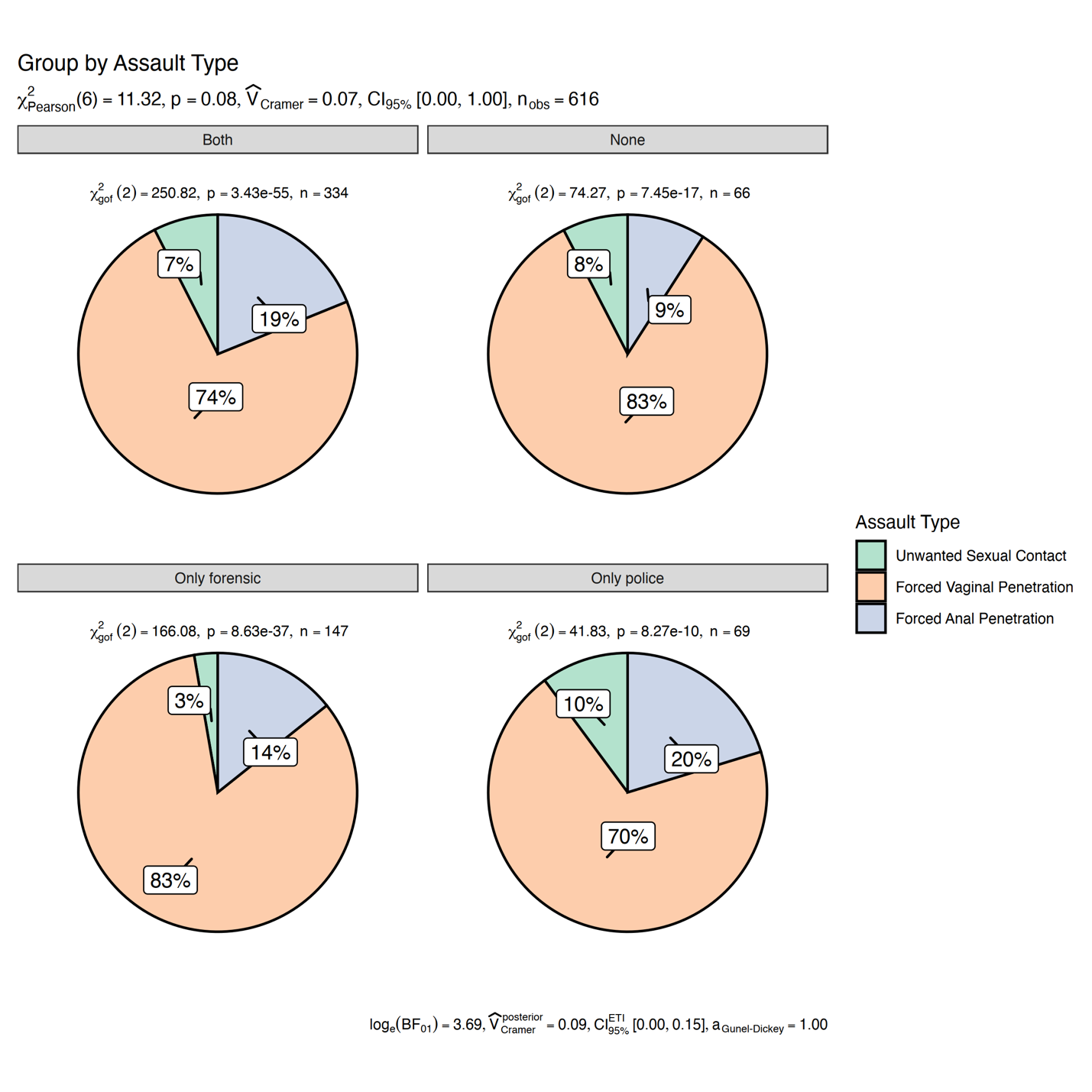


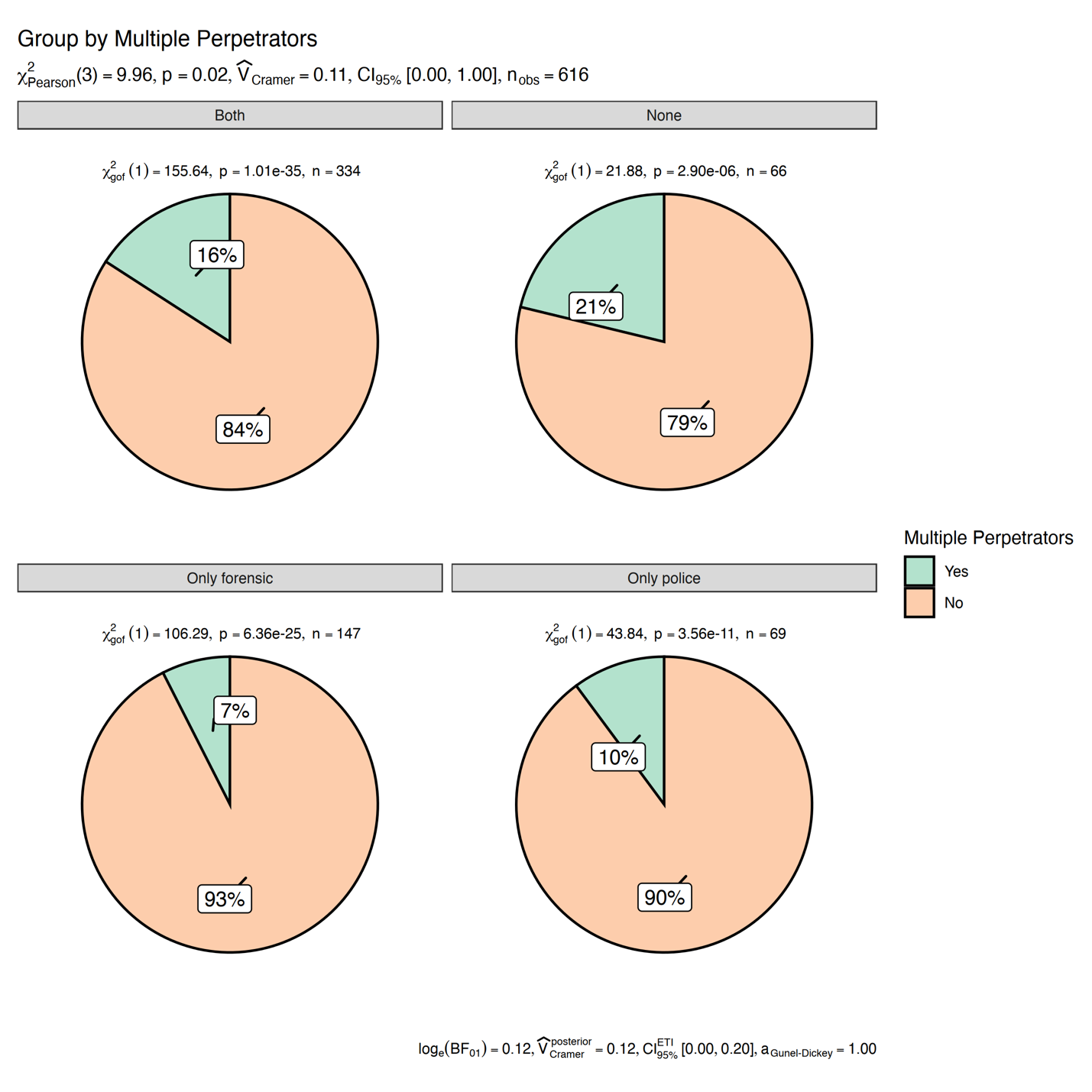


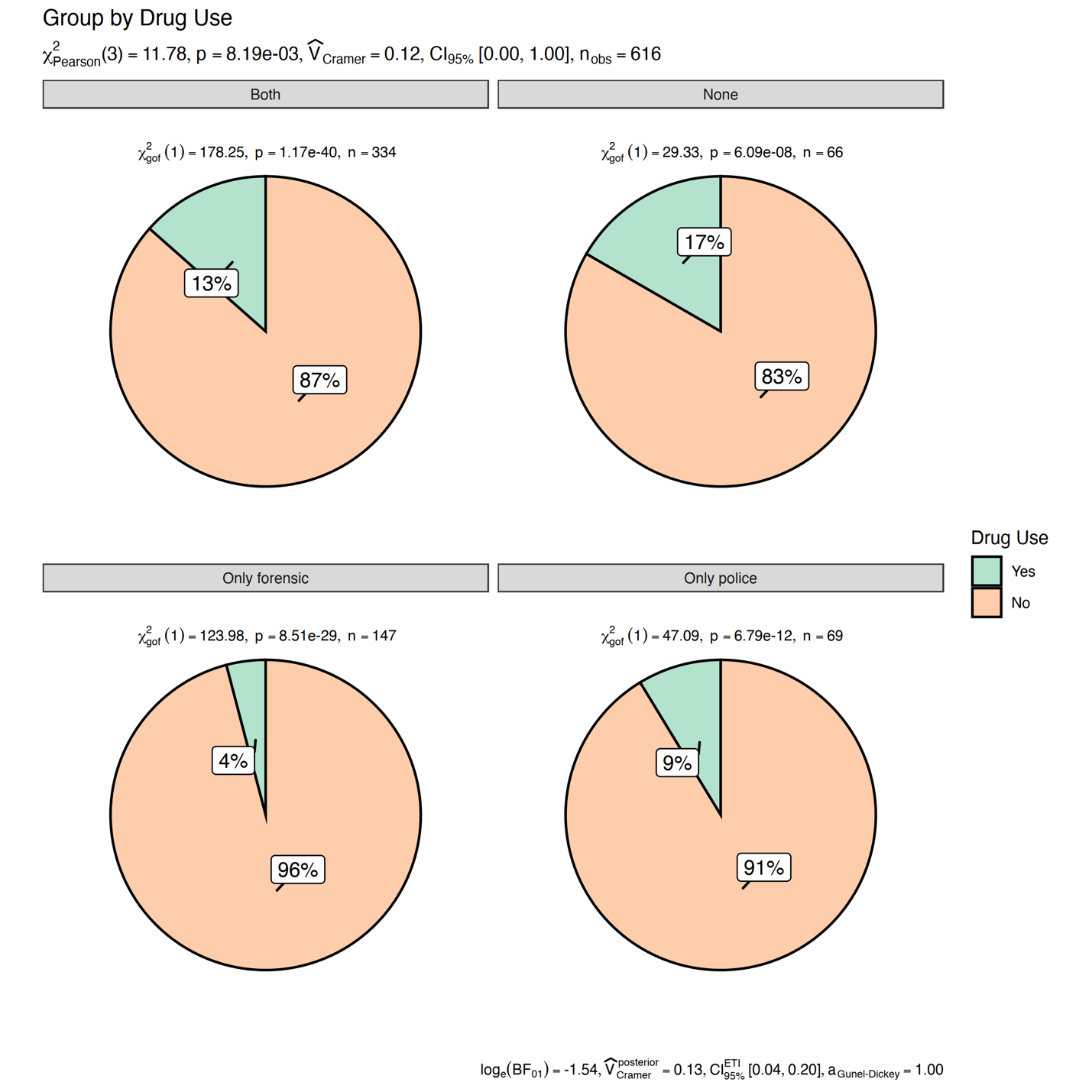


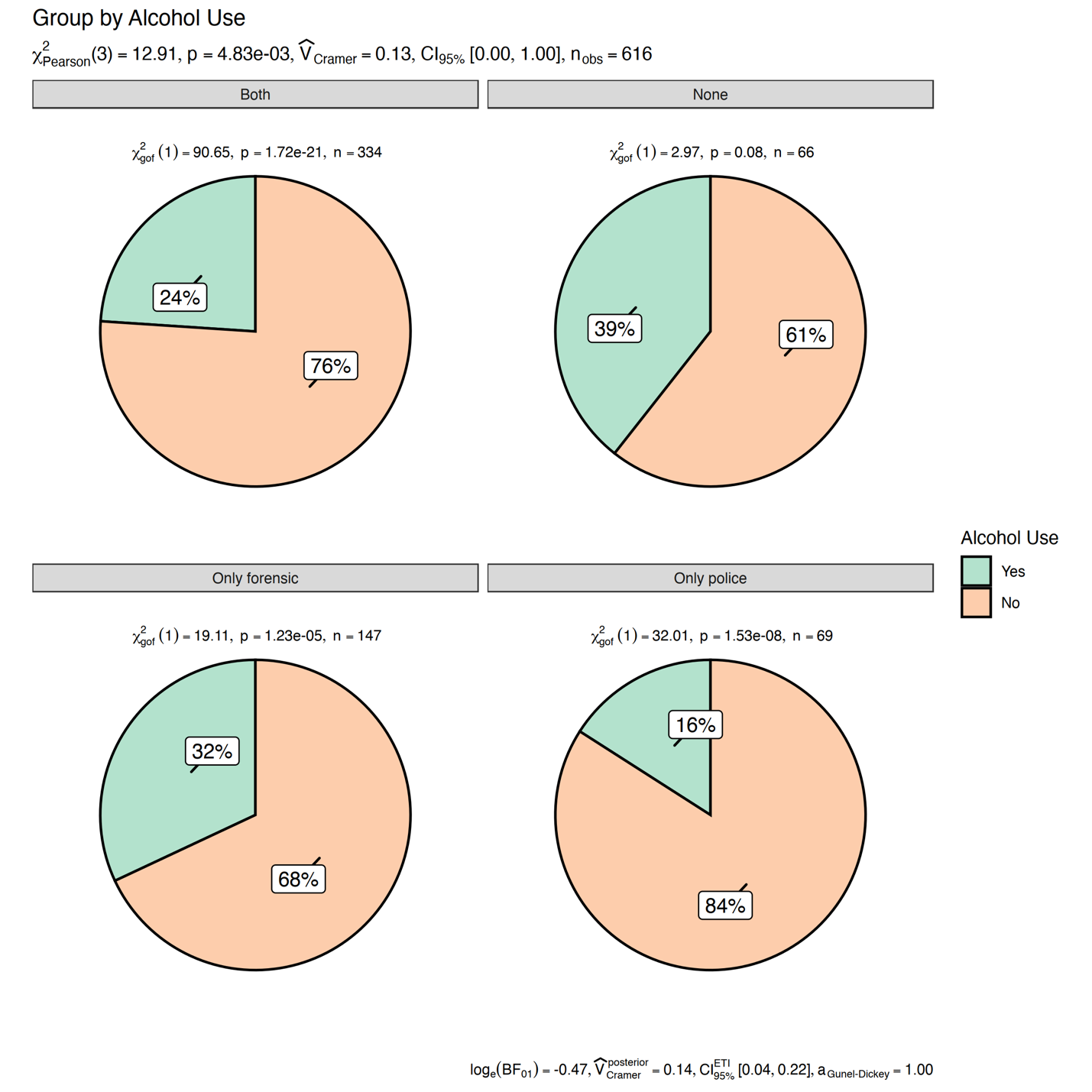


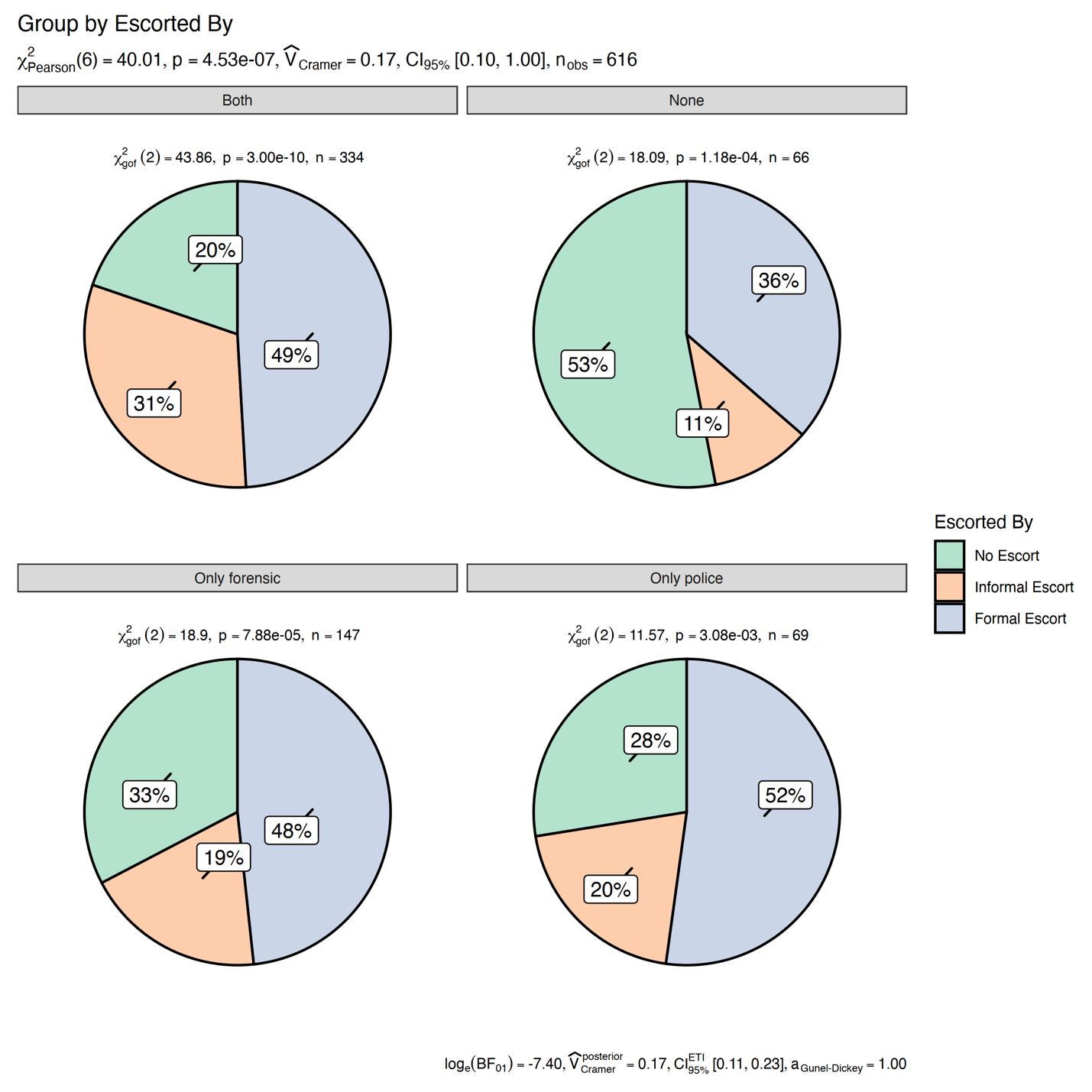


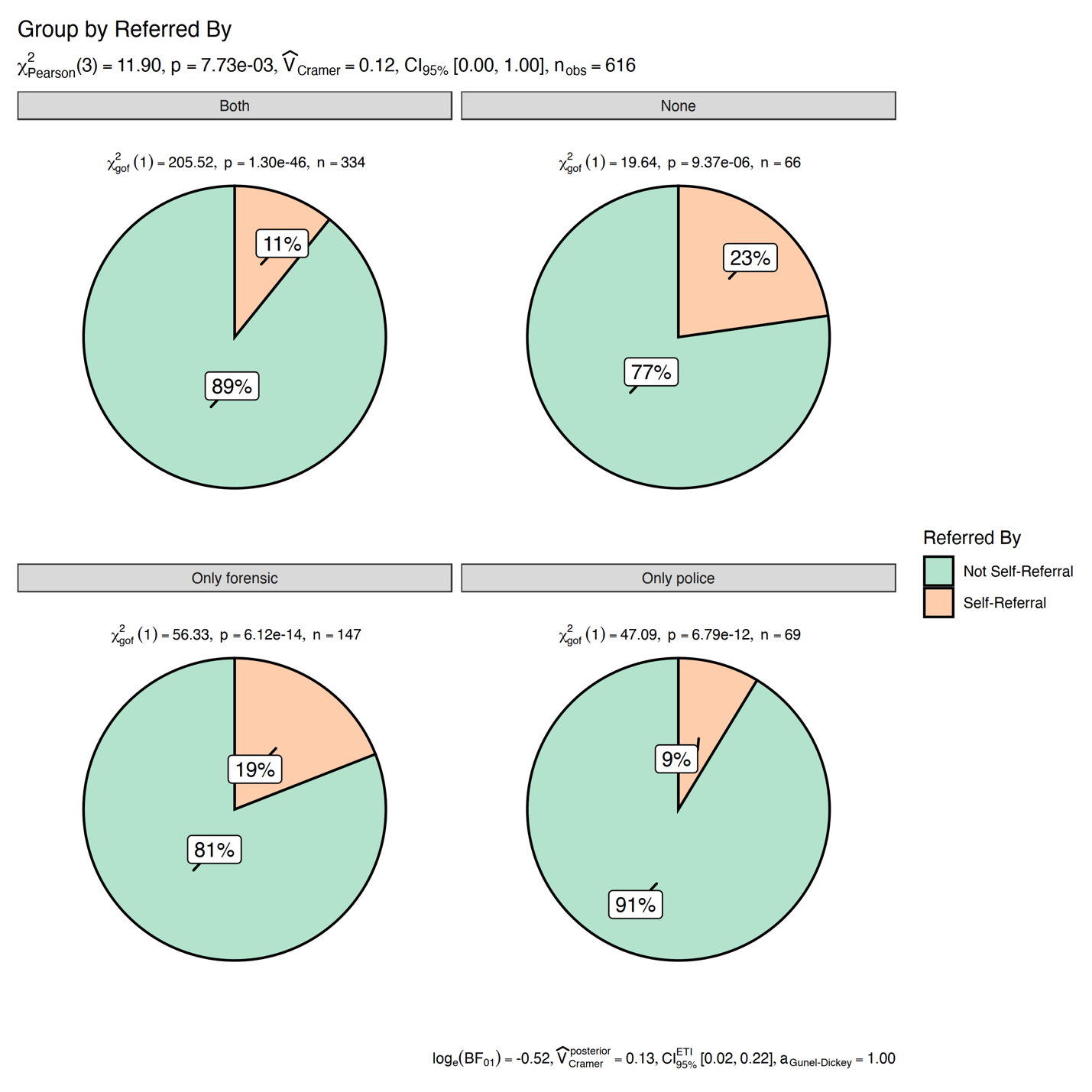

Supplement: Supplementary file 2 — Additional file 2. [file 13584_2025_697_MOESM2_ESM.docx]
