# Supplementary material for: Tailoring care, advancing justice: predictors of forensic and legal engagement in survivors of sexual violence
Source: Isr J Health Policy Res. 2025 Jun 23;14:38. doi: 10.1186/s13584-025-00697-1 (PMC12183890; doi:10.1186/s13584-025-00697-1)
Supplement: Supplementary file 3 — Additional file 3. [file 13584_2025_697_MOESM3_ESM.doc]

| **Table 2 – Results of Multinomial Ordinal Regression Model** | **Group** | | | |
| --- | --- | --- | --- | --- |
| *Predictors* | *Odds Ratios* | *CI* | *p* | *Comparison* |
| (Intercept) | 14.89 | 0.97 – 228.90 | 0.053 | None-Both |
| Time since the assault | 0.81 | 0.70 – 0.93 | **0.004** | None-Both |
| Biological sex (Women) | 0.07 | 0.01 – 0.67 | **0.022** | None-Both |
| Ethnicity (Arabic) | 1.47 | 0.65 – 3.28 | 0.352 | None-Both |
| Assault type: Unwanted sex vs. Forced vaginal | 0.22 | 0.06 – 0.77 | **0.018** | None-Both |
| Assault type: Unwanted sex vs. Forced anal | 0.21 | 0.05 – 0.88 | **0.033** | None-Both |
| Multiple perpetrators | 0.62 | 0.28 – 1.41 | 0.255 | None-Both |
| Drug use | 0.96 | 0.42 – 2.24 | 0.931 | None-Both |
| Alcohol use | 0.41 | 0.21 – 0.78 | **0.007** | None-Both |
| Escorted: No vs. Informal | 1.99 | 0.97 – 4.09 | 0.062 | None-Both |
| Escorted: No vs. Formal | 4.45 | 1.72 – 11.51 | **0.002** | None-Both |
| Referred by (Non-self referral) | 1.50 | 0.66 – 3.43 | 0.337 | None-Both |
| Medication (yes) | 3.43 | 1.70 – 6.91 | **0.001** | None-Both |
|  |  |  |  |  |
| (Intercept) | 6.63 | 0.38 – 115.97 | 0.194 | None-Only forensic |
| Time since the assault | 1.07 | 0.95 – 1.21 | 0.255 | None-Only forensic |
| Biological sex (Women) | 0.12 | 0.01 – 1.29 | 0.080 | None-Only forensic |
| Ethnicity (Arabic) | 0.84 | 0.34 – 2.03 | 0.692 | None-Only forensic |
| Assault type: Unwanted sex vs. Forced vaginal | 0.43 | 0.11 – 1.71 | 0.229 | None-Only forensic |
| Assault type: Unwanted sex vs. Forced anal | 0.35 | 0.07 – 1.67 | 0.189 | None-Only forensic |
| Multiple perpetrators | 0.28 | 0.11 – 0.74 | **0.010** | None-Only forensic |
| Drug use | 0.27 | 0.09 – 0.84 | **0.023** | None-Only forensic |
| Alcohol use | 0.60 | 0.30 – 1.18 | 0.136 | None-Only forensic |
| Escorted: No vs. Informal | 1.65 | 0.77 – 3.52 | 0.195 | None-Only forensic |
| Escorted: No vs. Formal | 1.96 | 0.72 – 5.35 | 0.187 | None-Only forensic |
| Referred by (Non-self referral) | 0.99 | 0.42 – 2.33 | 0.981 | None-Only forensic |
| Medication (yes) | 4.14 | 1.93 – 8.86 | **<0.001** | None-Only forensic |
|  |  |  |  |  |
| (Intercept) | 19.47 | 1.13 – 335.32 | **0.041** | None-Only police |
| Time since the assault | 0.99 | 0.87 – 1.12 | 0.823 | None-Only police |
| Biological sex (Women) | 0.07 | 0.01 – 0.77 | **0.029** | None-Only police |
| Ethnicity (Arabic) | 0.72 | 0.27 – 1.92 | 0.509 | None-Only police |
| Assault type: Unwanted sex vs. Forced vaginal | 0.38 | 0.10 – 1.37 | 0.138 | None-Only police |
| Assault type: Unwanted sex vs. Forced anal | 0.34 | 0.07 – 1.55 | 0.162 | None-Only police |
| Multiple perpetrators | 0.39 | 0.13 – 1.18 | 0.095 | None-Only police |
| Drug use | 0.61 | 0.19 – 1.94 | 0.399 | None-Only police |
| Alcohol use | 0.29 | 0.12 – 0.69 | **0.005** | None-Only police |
| Escorted: No vs. Informal | 2.72 | 1.14 – 6.47 | **0.024** | None-Only police |
| Escorted: No vs. Formal | 3.83 | 1.26 – 11.65 | **0.018** | None-Only police |
| Referred by (Non-self referral) | 1.87 | 0.60 – 5.79 | 0.280 | None-Only police |
| Medication (yes) | 0.79 | 0.33 – 1.88 | 0.590 | None-Only police |
|  |  |  |  |  |
| (Intercept) | 0.44 | 0.08 – 2.63 | 0.371 | Both-Only forensic |
| Time since the assault | 1.32 | 1.17 – 1.50 | **<0.001** | Both-Only forensic |
| Biological sex (Women) | 1.77 | 0.66 – 4.74 | 0.253 | Both-Only forensic |
| Ethnicity (Arabic) | 0.57 | 0.32 – 1.03 | 0.064 | Both-Only forensic |
| Assault type: Unwanted sex vs. Forced vaginal | 1.96 | 0.66 – 5.83 | 0.228 | Both-Only forensic |
| Assault type: Unwanted sex vs. Forced anal | 1.69 | 0.51 – 5.61 | 0.393 | Both-Only forensic |
| Multiple perpetrators | 0.45 | 0.22 – 0.93 | **0.032** | Both-Only forensic |
| Drug use | 0.28 | 0.11 – 0.71 | **0.008** | Both-Only forensic |
| Alcohol use | 1.47 | 0.92 – 2.36 | 0.110 | Both-Only forensic |
| Escorted: No vs. Informal | 0.83 | 0.49 – 1.40 | 0.484 | Both-Only forensic |
| Escorted: No vs. Formal | 0.44 | 0.25 – 0.79 | **0.006** | Both-Only forensic |
| Referred by (Non-self referral) | 0.66 | 0.35 – 1.23 | 0.190 | Both-Only forensic |
| Medication (yes) | 1.21 | 0.72 – 2.03 | 0.477 | Both-Only forensic |
|  |  |  |  |  |
| (Intercept) | 1.31 | 0.20 – 8.36 | 0.778 | Both-Only police |
| Time since the assault | 1.22 | 1.06 – 1.40 | **0.005** | Both-Only police |
| Biological sex (Women) | 1.06 | 0.35 – 3.21 | 0.918 | Both-Only police |
| Ethnicity (Arabic) | 0.49 | 0.23 – 1.06 | 0.070 | Both-Only police |
| Assault type: Unwanted sex vs. Forced vaginal | 1.73 | 0.62 – 4.77 | 0.292 | Both-Only police |
| Assault type: Unwanted sex vs. Forced anal | 1.60 | 0.47 – 5.47 | 0.449 | Both-Only police |
| Multiple perpetrators | 0.63 | 0.24 – 1.63 | 0.337 | Both-Only police |
| Drug use | 0.63 | 0.23 – 1.75 | 0.376 | Both-Only police |
| Alcohol use | 0.71 | 0.33 – 1.52 | 0.375 | Both-Only police |
| Escorted: No vs. Informal | 1.37 | 0.67 – 2.80 | 0.393 | Both-Only police |
| Escorted: No vs. Formal | 0.86 | 0.39 – 1.89 | 0.711 | Both-Only police |
| Referred by (Non-self referral) | 1.25 | 0.45 – 3.47 | 0.675 | Both-Only police |
| Medication (yes) | 0.23 | 0.12 – 0.44 | **<0.001** | Both-Only police |
|  |  |  |  |  |
| (Intercept) | 2.93 | 0.37 – 23.05 | 0.305 | Only forensic-Only police |
| Time since the assault | 0.92 | 0.82 – 1.03 | 0.151 | Only forensic-Only police |
| Biological sex (Women) | 0.60 | 0.16 – 2.21 | 0.439 | Only forensic-Only police |
| Ethnicity (Arabic) | 0.86 | 0.36 – 2.05 | 0.732 | Only forensic-Only police |
| Assault type: Unwanted sex vs. Forced vaginal | 0.88 | 0.25 – 3.07 | 0.845 | Only forensic-Only police |
| Assault type: Unwanted sex vs. Forced anal | 0.95 | 0.22 – 4.04 | 0.947 | Only forensic-Only police |
| Multiple perpetrators | 1.40 | 0.47 – 4.19 | 0.551 | Only forensic-Only police |
| Drug use | 2.22 | 0.63 – 7.83 | 0.213 | Only forensic-Only police |
| Alcohol use | 0.48 | 0.22 – 1.07 | 0.072 | Only forensic-Only police |
| Escorted: No vs. Informal | 1.65 | 0.77 – 3.52 | 0.198 | Only forensic-Only police |
| Escorted: No vs. Formal | 1.95 | 0.83 – 4.60 | 0.125 | Only forensic-Only police |
| Referred by (Non-self referral) | 1.89 | 0.65 – 5.44 | 0.240 | Only forensic-Only police |
| Medication (yes) | 0.19 | 0.09 – 0.40 | **<0.001** | Only forensic-Only police |
| Observations | 616 | | | |
| R^2^ / R^2^ adjusted | 0.159 / 0.157 | | | |

**Figure 1.** The likelihood of being a member of each study group as a function of the time since the assault.

**
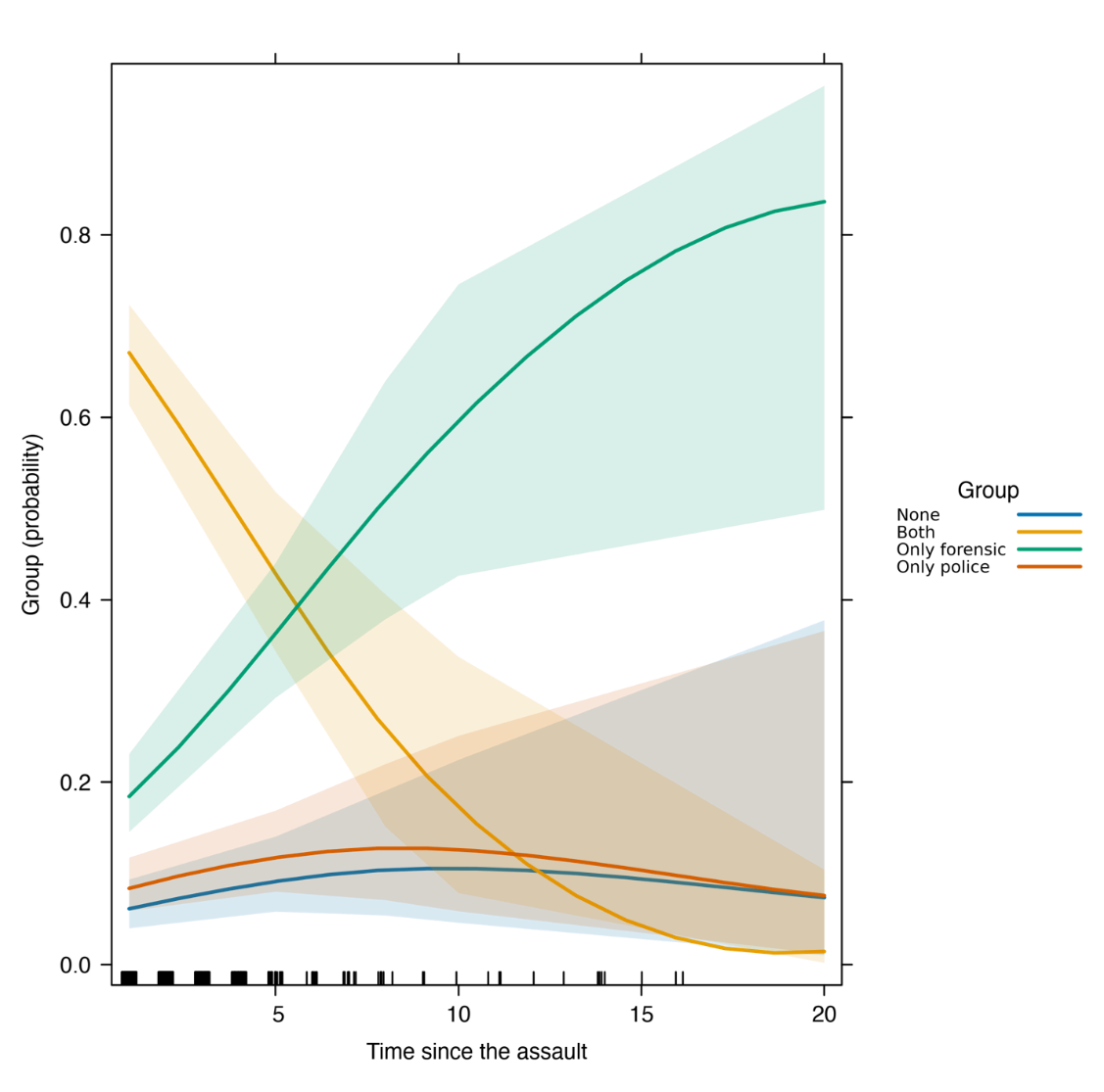
**

Figure 1 caption: The probability of belonging to each study group ("None," "Both," "Only forensic," and "Only police") as a function of the time since the assault. The y-axis represents the likelihood of group membership, while the x-axis indicates the time elapsed since the assault (in days). The shaded regions around each line represent the confidence intervals.


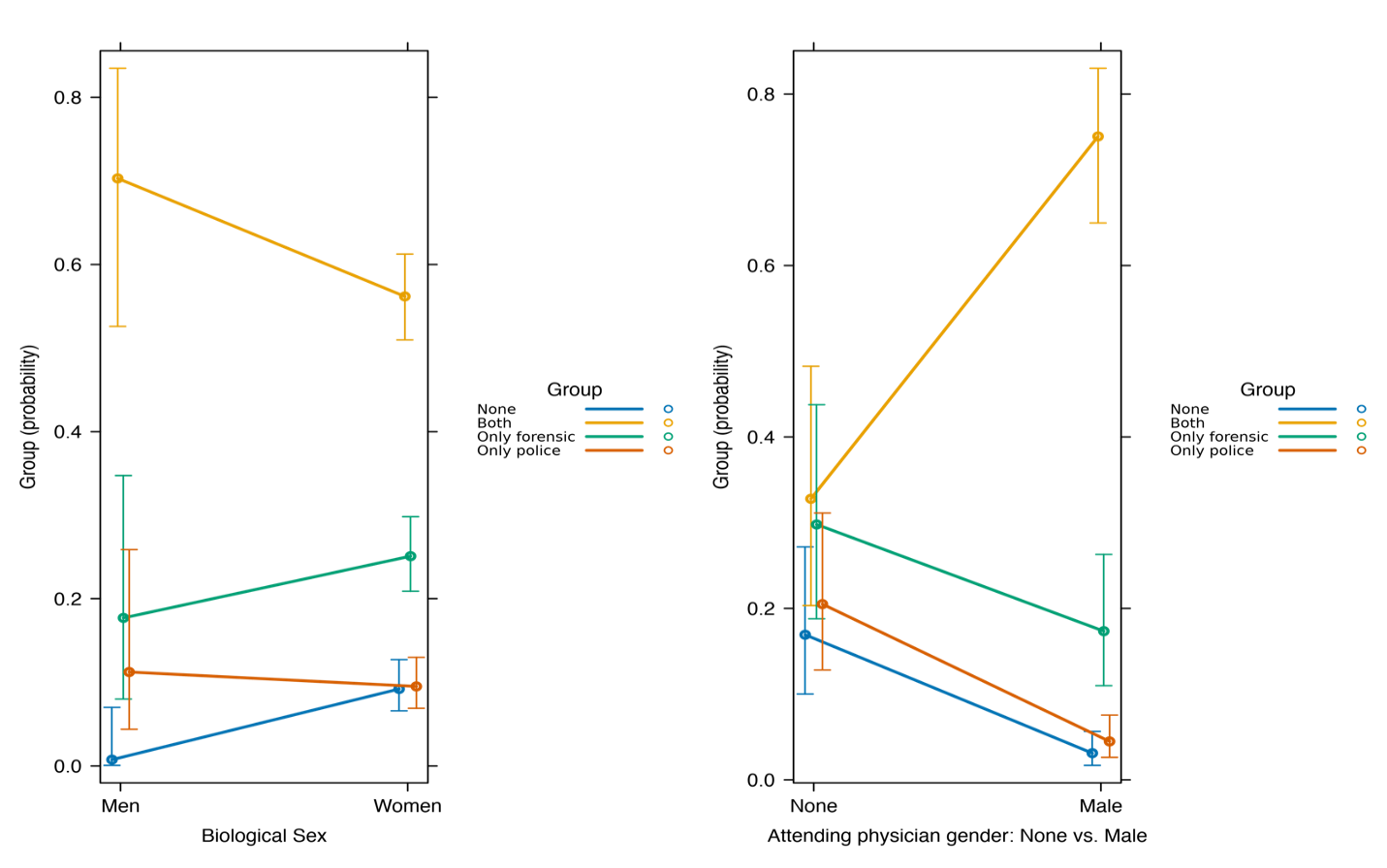
**Figure 2.** The likelihood of being a member of each study group as a function of victims’ biological sex.

Figure 2 caption: The probability of belonging to each study group ("None," "Both," "Only forensic," and "Only police") as a function of biological sex. The y-axis represents the likelihood of group membership, while the x-axis indicates the gender of the survivor.

**Figure 3.** The likelihood of being a member of each study group as a function of the presence of multiple perpetrators.


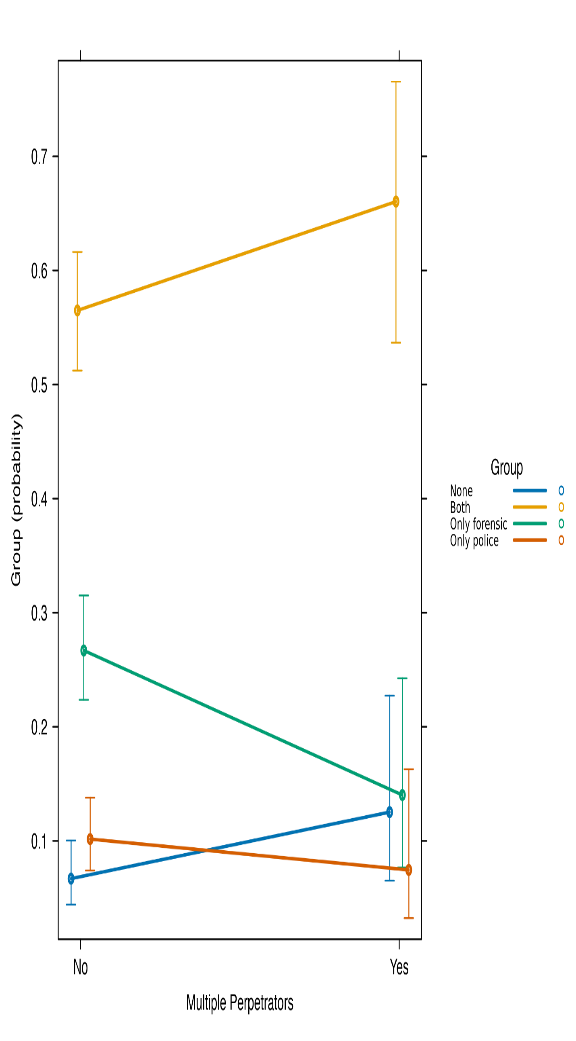


Figure 3 caption: The probability of belonging to each study group ("None," "Both," "Only forensic," and "Only police") as a function of the presence of multiple perpetrators. The y-axis represents the likelihood of group membership, while the x-axis indicates the whether there were multiple perpetrators (yes or no).

**Figure 4.** The likelihood of being a member of each study group as a function of the assault type.

Assault type: Unwanted sex vs. Forced anal

Assault type: Unwanted sex vs. Forced vaginal


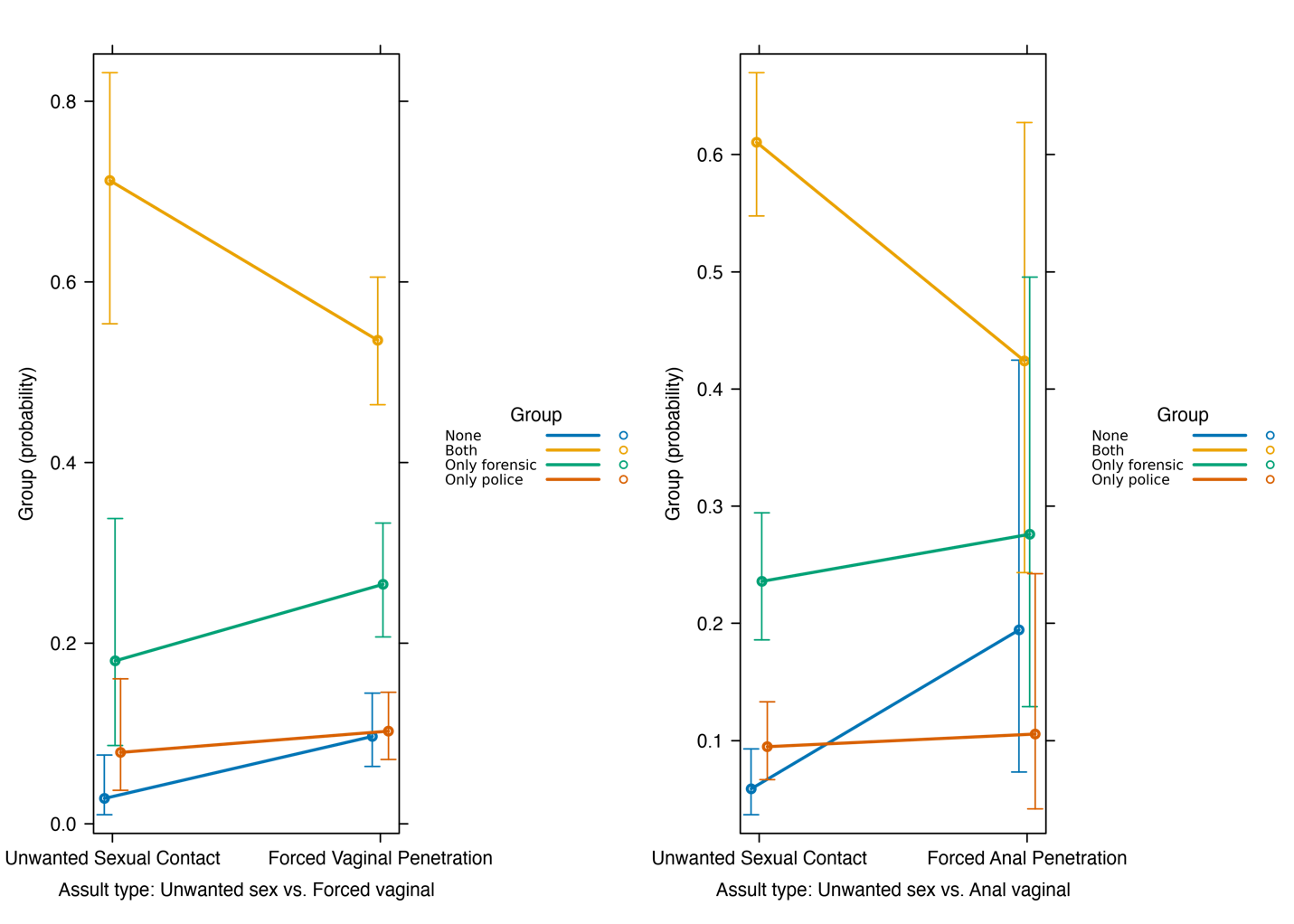


Figure 4 caption: The probability of belonging to each study group ("None," "Both," "Only forensic," and "Only police") as a function of assault type; unwanted sex vs. forced vaginal penetration (left figure) and unwanted sex vs. forced anal penetration (right figure). The y-axis represents the likelihood of group membership, while the x-axis indicates the assault type.

**Figure 5.** The likelihood of being a member of each study group as a function of alcohol use.


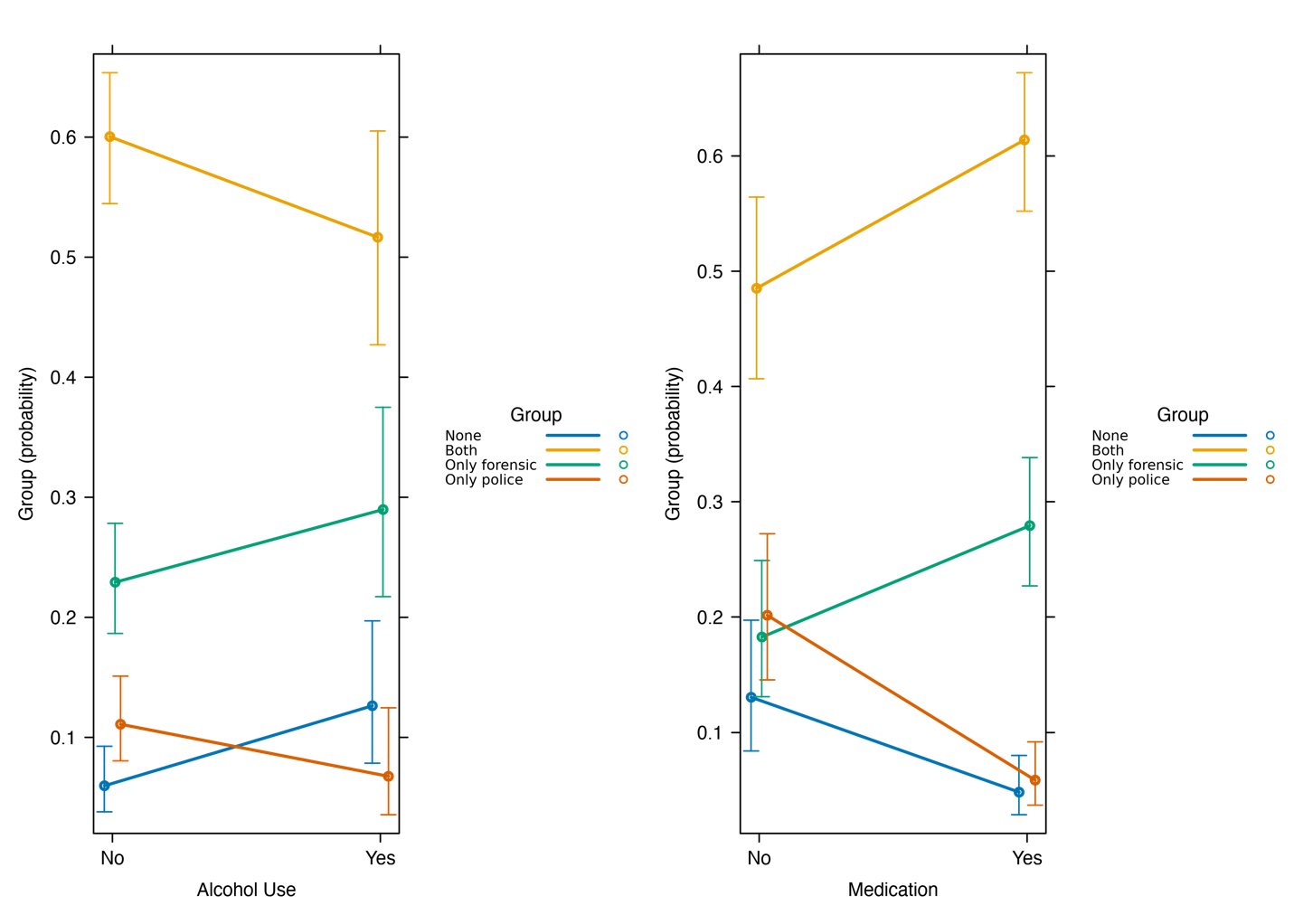


Figure 5 caption: The probability of belonging to each study group ("None," "Both," "Only forensic," and "Only police") as a function of alcohol use. The y-axis represents the likelihood of group membership, while the x-axis indicates use of alcohol.

**Figure 6.** The likelihood of being a member of each study group as a function of survivor's consent to receive medication.


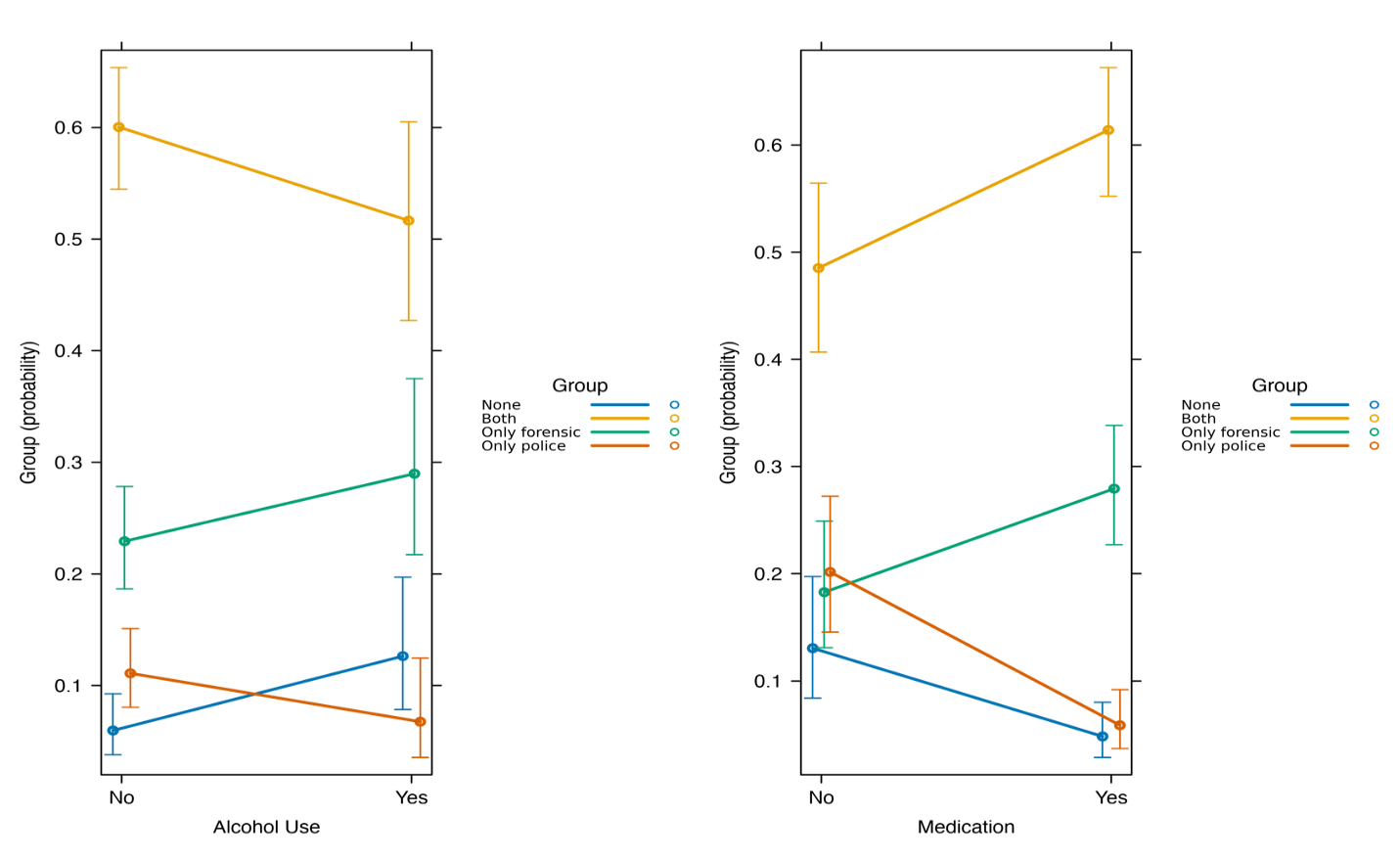


Figure 6 caption: The probability of belonging to each study group ("None," "Both," "Only forensic," and "Only police") as a function of survivor's consent to receive medication. The y-axis represents the likelihood of group membership, while the x-axis indicates consent to receive medication (yes or no).

**Figure 7.** The likelihood of being a member of each study group as a function of who escorted the victim (no escort vs. informal escort).


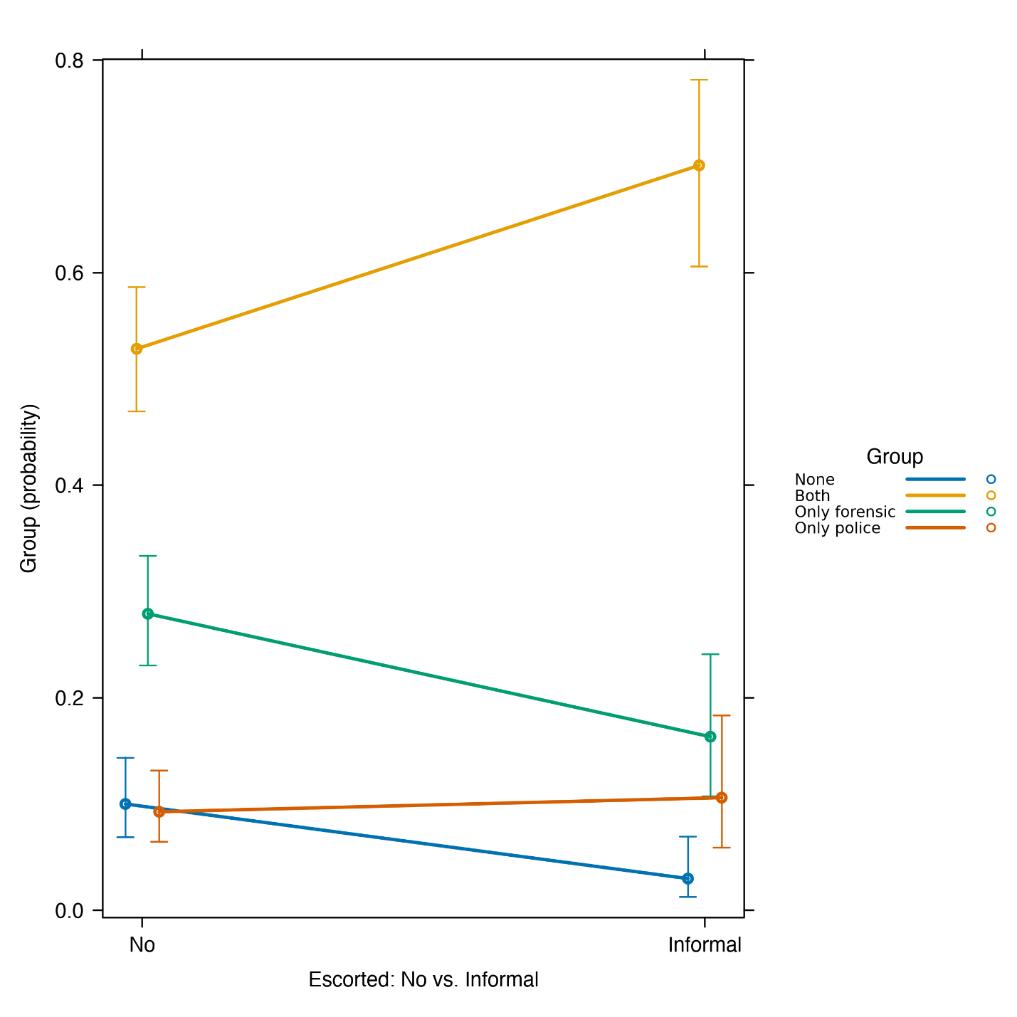


Figure 7 caption: The probability of belonging to each study group ("None," "Both," "Only forensic," and "Only police") as a function of who escorted the victim. The y-axis represents did the survivor arrived unaccompanied or with an informal escort (family or friends).
